# Explanatory model of emotional-cognitive variables in school mathematics performance: a longitudinal study in primary school

**DOI:** 10.3389/fpsyg.2015.01363

**Published:** 2015-09-08

**Authors:** Gamal Cerda, Carlos Pérez, José I. Navarro, Manuel Aguilar, José A. Casas, Estíbaliz Aragón

**Affiliations:** ^1^Departamento de Metodología de la Investigación e Informática Educacional, Facultad de Educación, Universidad de ConcepciónConcepción, Chile; ^2^Departamento de Ingeniería Matemática, Facultad de Ciencias Físicas y Matemáticas, Universidad de ConcepciónConcepción, Chile; ^3^Departamento de Psicología, Universidad de CádizPuerto Real, Spain; ^4^Departamento de Psicología, Universidad de CórdobaCórdoba, Spain

**Keywords:** mathematical competencies, logical intelligence, structural model, predisposition, performance

## Abstract

This study tested a structural model of cognitive-emotional explanatory variables to explain performance in mathematics. The predictor variables assessed were related to students’ level of development of early mathematical competencies (EMCs), specifically, relational and numerical competencies, predisposition toward mathematics, and the level of logical intelligence in a population of primary school Chilean students (*n* = 634). This longitudinal study also included the academic performance of the students during a period of 4 years as a variable. The sampled students were initially assessed by means of an Early Numeracy Test, and, subsequently, they were administered a Likert-type scale to measure their predisposition toward mathematics (EPMAT) and a basic test of logical intelligence. The results of these tests were used to analyse the interaction of all the aforementioned variables by means of a structural equations model. This combined interaction model was able to predict 64.3% of the variability of observed performance. Preschool students’ performance in EMCs was a strong predictor for achievement in mathematics for students between 8 and 11 years of age. Therefore, this paper highlights the importance of EMCs and the modulating role of predisposition toward mathematics. Also, this paper discusses the educational role of these findings, as well as possible ways to improve negative predispositions toward mathematical tasks in the school domain.

## Introduction

Predictors of academic performance can be classified into two differentiated, but related categories: the first category contains domain-general predictor abilities and the second one contains domain-specific predictor abilities (Passolunghi and Lanfranchi, 2012). Among domain-general predictors, we can find cognitive and emotional abilities, which predict performance in the wide spectrum of school subjects and not just on a particular content domain, like, for example, general intelligence or motivation. In contrast, specific predictors concern abilities that predict future performance in particular fields, like number-sense or counting abilities in mathematics (De Smedt et al., 2009). This research will examine both domain-general and domain-specific predictors. The domain-general predictors to be examined are the role of logical intelligence and predisposition toward mathematics. As for domain-specific predictors, the role of logical and relational types of early mathematical competencies (EMCs) and numerical competencies will be examined.

In Chile, the Piagetian educational theories have had a strong influence in the field of school instruction, especially in the areas related to the process of number development. According to Piaget and Szeminska (1941), coordinating operations of seriation and classification allows children to comprehend numbers as a set of classes that are categorized in an organized manner. However, Piaget’s proposed age ranges for children’s developmental stages and the expected full internalization of the operations associated with these stages have been revised because individuals show high variability in their specific development (Fuchs et al., 2006; Kamii and Russell, 2010). In addition, the Piagetian approach seems to underestimate the role of language and counting in the development of number competencies (Barrouillet and Camos, 2002). Research with babies carried out by McCrink and Wynn (2009) shows evidence that the subjects’ capacity to understand number concepts seem to appear earlier than proposed by Piaget. Equally, number comprehension is developed gradually through counting experience (Lehalle, 2002), which indicates a more complex process than the simple recollection of numerical sequences (Gelman and Butterworth, 2005). Along this same line, there are authors who point out that the basis of mathematical development is logical thought, even though it may seem that the contribution of counting is greater than the contribution of logical operations (Bryant and Nunes, 2009).

This research has adopted an interactionist approach, which considers that Piagetian operations and counting do not occur in a separate and sequential manner, but that they both contribute to the comprehension of number concepts. Thus, the development of number concepts has been reformulated into a construct, namely EMC, as researchers believe that an appropriate conceptualization of numbers necessarily involves the use of logical properties and counting capacities (Van Luit et al., 1998). Thereby, the Piagetian approach, which is currently the dominant theory of number development in the Chilean educational system, is combined with other perspectives in this study, in order to accomplish a better comprehension of mathematical competencies, especially, numerical ones (Van Luit, 2006) in the Chilean context.

Several longitudinal studies have found evidence that, irrespective from sociodemographic factors, counting acquisition and relational abilities before formal schooling are highly predictive factors for the acquisition of basic arithmetical abilities, as well as for overall mathematics performance in early school levels (Byrnes and Wasik, 2009; Aunio and Niemivirta, 2010). Likewise, Jordan et al. (2009) posit that having a high level of mathematical competence in early education significantly predicts subsequent mathematics accomplishments until the third year of primary school. Furthermore, research conducted by Mazzocco and Thompson (2005) and Locuniak and Jordan (2008) report that EMCs predict results about general cognitive competencies even beyond the field of mathematics, like verbal competencies, spatial competencies, and memory skills. In Chile, the results of the application of a scale to assess EMCs, the Early Numeracy Test (ENT), showed results in line with other international results reported, which confirm that students perform better in tasks involving logical-relational competencies over tasks involving numerical competencies. Furthermore, when comparing groups according to gender, the results from the application of this scale showed no significant differences based on gender (Cerda et al., 2014). As in other countries, these findings allow us to warn the scientific and academic communities about possible mathematics learning deficits in children, which are illuminated by attempts to solve counting problems and Piagetian tasks (Navarro et al., 2012).

In addition, there is consensus in the scientific community about the multitude of variables and factors that can explain overall variability in school performance. Many scientific studies have highlighted the existence of a complex and interdependent relationship between cognitive and emotional-attitudinal factors. Specifically, research has focused on analyzing the processes of activation, acquisition, and development of school knowledge and how they affect the incidence of positive academic performance (McKenzie et al., 2004; Miñano and Castejón, 2011).

The studies about mathematics learning and performance in mathematics have also followed this direction, and an important number of studies emphasize the interdependence of variables such as attitude toward the school subject, fluid intelligence, and academic motivation, as factors for achievement in mathematics (Moenikia and Zahed-Babelan, 2010; Suárez-Álvarez et al., 2014). Other studies have pointed out the close relationship between emotional factors and performance in mathematics (Pinxten et al., 2014). As a matter of fact, several studies have detected high levels of interdependence between anxiety, attitude, and self-efficacy with respect to academic success or failure in mathematics (Morony et al., 2013; Núñez-Peña et al., 2013).

On the other hand, logical intelligence is a cognitive ability that enables appropriate reasoning, like differentiating between a correct argument and an incorrect one, drawing inferences from comparing particular cases with the norm or a general law that rules them or extracting general conclusions from a set of specific propositions. The relationship between this type of inductive reasoning and academic performance is especially relevant (Klauer and Phye, 2008; Barkl et al., 2012), because students who show high levels of logical intelligence tend to not only have good academic performance in mathematics, but also have good overall academic performance (Cerda et al., 2011). Likewise, students that perform successfully in logical tasks, especially seriation, tend to perform best at solving arithmetical tasks (Aguilar et al., 2002; Navarro et al., 2011).

This correlation between performance in logical tasks and mathematics performance is due to the fact that these students show high abilities for organizing and recollecting relevant and situational information, which is a fundamental part of problem-solving (Rosselli et al., 2009). In relation to this, Taub et al. (2008) reported that fluid intelligence had a significant direct effect on mathematical performance, because students who possess this type of intelligence use strategies that influence directly on mathematics achievement, especially on tasks of inductive reasoning, which is the most determinant factor for the latent construct of fluid intelligence. In connection to the latter, recent results also reaffirm the existence of a relationship between mathematics performance and fluid intelligence (Gullick et al., 2011; Östergren and Träff, 2013).

Due to all the above, it is of great interest when researching about the teaching and learning of school mathematics to find predictors for students’ future academic performance. In this respect, this study aimed to find a model that is able to include both domain-general and domain-specific abilities (Guven and Cabakcor, 2013). Thus, the first objective of this research was to determine the predictive power of Piagetian, or logical-relational, type competencies and numerical competencies on EMCs in relation to the variability of school performance in a group of students (*n* = 634) during a period of 4 years. Accordingly, a second objective of this study was to create a model based on structural equations that could be parsimonious enough to explain the role and interaction of these variables on mathematics performance. This model drew data from the students’ early assessment of mathematical competencies along with students’ assessed level of inductive logical reasoning and predisposition toward mathematics.

### Hypotheses

H1: The exogenous variable concerning EMCs of logical-relational type and numerical type (which was initially assessed) will have a positive effect on students’ academic performance in mathematics through the 4-years period under review.
H2: The variable *unfavorable predisposition toward mathematics* will negatively influence students’ performance in mathematics in the 4-years period under review.
H3: The variable *logical intelligence* will have a positive effect on students’ performance in mathematics in the 4-years period under review.
H4: The variables relational and numerical mathematical competences will be positively related to the level of logical intelligence and negatively related to an unfavorable predisposition toward mathematics.

In sum, our research hypotheses were that the EMCs variable along with the predisposition toward mathematics variable will highly influence students’ performance in mathematics. Secondly, we posit that mathematics competencies of logical-relational type will have a higher predictive power than numerical type competencies when assessing both these competencies separately.

## Materials and Methods

### Participants

The participants attended Chilean schools and their ages ranged from 8 to 11 years of age. In respect to the participants, it is important to note that the levels of the school education system in Chile are the following: preschool level: preschool is non-compulsory and is divided into two levels; it is for children up to 5 years of age; Primary school: primary school is compulsory and is divided into 8 different levels; it is for children up to 13 years of age; Secondary school: secondary school is compulsory and is divided into four different levels; it is for children up to 18 years of age.

In terms of type of school dependencies, the following type of schools can be found in the Chilean school system: *Municipal* (public schools run by city governments); *Particular subvencionada* (private-subsidized schools); and *Particular* (Private non-subsidized schools). Most Chilean students attend private-subsidized schools.

In this particular study, the participants were 634 primary school Chilean students (51.6% male), who ranged in age from 8 to 11 (*M* = 9.93, *SD* = 0.937). These students were enrolled in primary school courses ranging from third grade to sixth grade. A non-probabilistic accidental sampling method was used to obtain the sample. The sampled students were selected from the group of students that had already had their level of EMCs previously assessed and that were still enrolled at the same educational centers 4 years after the initial assessment. Since the students were all underage, their parents individually accepted informed consent before starting the study and they were told that they could ask any question, any time during the process.

### Instruments

#### Early Numeracy Test

A version of the ENT (Van Luit et al., 1998) that was previously adapted for use in Chile (Cerda et al., 2012) was used. This test is used to assess early numeric knowledge as well to detect students with learning difficulties in mathematics. ENT was composed of three parallel versions of 40 items each, and each version of the test had a maximum score of 40 points (one point for each correct item). The average ENT test application period was ∼30 min, and it was administered individually to each student. The test assessed eight components of EMCs: comparison concepts, classification, one-to-one correspondence, seriation, verbal counting, structured counting, resultative counting (counting without the need to point or touch) and general number knowledge. The Cronbach’s alpha was 0.90.

#### Basic Test of Logical Intelligence (TILE)

This test was standardized in Chile and has different scales according to age and social extraction (Cerda et al., 2010). Test of Logical Intelligence (TILE) is a figurative type of test composed of 50 incomplete series, and the amount of time allowed for answering is controlled. Each series has four geometrical figures that are linked by a pattern. To complete the series correctly, the test subject must choose among the five given possibilities. TILE had a maximum score of 50 points (one point for each correct item). The Cronbach’s alpha was 0.94.

#### Predisposition toward Mathematics Scale (EPMAT)

The original instrument (CAT-Ma) was designed and validated with a Spanish sample by Del Rey et al. (2011) and had 13 items that examined three dimensions: self-confidence, resilience, and emotional block. The Chilean version, validated by Cerda et al. (in press a), has six items that examine a single factor, namely predisposition toward mathematics, which refers to the degree of predisposition toward mathematics contents. The six test items were as follows: “I know I will not be successful in mathematics,” “My results in mathematics have always been bad,” “I am not good at mathematics,” “I do not like mathematics,” “I can never solve mathematical problems,” and “Number operations are easy for me to solve.” EPMAT is a Likert scale with six items. The overall test score ranges from 6 to 30 points. The Cronbach’s alpha was 0.82.

#### Academic Performance in Mathematics

In this study, academic performance was operationalized as the grade point average obtained each trimester by the sampled students in the subject of mathematics during the 4-years period under review. In Chile, schools have the obligation of recording students’ grades and sending these transcripts to the Ministry of Education. After obtaining informed consent from the schools and the students’ parents, students’ transcripts were accessed by the research team. Transcripts, which included students’ gender, educational level, yearly-gpa and age, were transferred to spreadsheets for research purposes.

### Procedure

This study was carried out in accordance with a protocol written by the Chilean researchers. The “Protocol of Informed Consent,” was previously reviewed and approved by the Ethics Committee of the University of Concepción, Chile, and the study was carried out with written informed consent from all subjects. All subjects gave written informed consent in accordance with the Declaration of Helsinki.

Educational centers were contacted by means of an information letter, which established a protocol of informed consent. An individual from each academic center was designated as a responsible person for the procedure. This person was usually the teacher of the mathematics subject. The students took the test voluntarily and during school hours. All tests were administered by specialized staff under rigorous assessment criteria for psychological testing, according to the norms indicated by test administration guidelines.

Early Numeracy Test original version considers a total of 40 points. However, since the population considered in the study covers different age groups, and because the psycho-evolutive nature of the test, if we consider only raw scores, they do not adequately reflect the true performance of students based on their age, since, for instance, if a 4-years-old child solves three of the five tasks in one of the dimensions of the test, he could get the same score than a 8-years-old child when solving these same three tasks, but clearly the higher cognitive and educational level of the latter should enable it to reach more number of tasks. Thus, raw scores were standardized by age and educational level according to the normative scale. To avoid negative values, a math constant was added, allowing normalized scores remain statistically close to the original scale. This fact explains the existence of scores over five in the results section.

#### Data Analysis

To conduct the data analysis, we opted to represent the interaction between the different factors by means of a structural equations model. We employed a robust maximum-likelihood (RML) estimation method, due to the fact that the analyzed data was mainly ordinal (Flora and Curran, 2004). Also, following the recommendations from Hu and Bentler (1999), a combination of different indices was used to contrast the adequacy of the proposed models. Among these, we can name the chi- square statistic by Satorra and Bentler (2001), the comparative fit index (*CFI*), the non-normed fit index (*NNFI*), and the root mean square error of approximation (*RMSEA*). A model has optimal fitting when the ratio between the chi-square statistics are not significant, fit indices are equal or higher than 0.95 and *RMSEA* is under 0.05 (Jöreskog, 1994; Hu and Bentler, 1999).

Standardized regression coefficients included in the model were estimated by analyzing their level of significance and their compliance with the values of asymmetry and kurtosis (asymmetry <|2|; and kurtosis <|7|; West et al., 1995; see **Table 1**). Likewise, residual scatter plots show that the relationship between variables is linear, since the points have the same dispersion along all data values, and there is no presence of any regular or curvilinear forms that could indicate a possible absence of linearity or the existence of heteroscedasticity. Finally, a multivariate outlier diagnostic assessed with the Mahalanobis distance indicated that four observations concerning some dimensions of EMCs had values of signification under 0.001, which was established as the threshold value (Hair et al., 2005). Therefore, after verifying that the observations were not representative of a segment of the population and because the sample was large enough, we decided to delete the aforementioned observations. The data analysis was conducted with the statistical software EQS 6.2.

**Table 1:** Mean, standard deviation (*SD*), asymmetry, and kurtosis of the variables included in the models.

Variables	Mean	*SD*	Asymmetry	Kurtosis
EMC comparison	5.623	0.885	-1.812	2.732
EMC classification	5.563	0.935	-1.234	0.806
EMC correspondence	5.674	0.914	-0.999	0.836
EMC seriation	5.646	0.997	-0.756	0.602
EMC verbal counting	5.720	0.870	-1.190	1.759
EMC resultative counting	5.695	1.006	-0.102	0.097
EMC structured counting	5.760	1.052	-0.286	0.532
EMC general number knowledge	5.708	0.956	-1.005	1.497
Logical intelligence	26.810	11.560	-0.095	-0.951
Predisposition toward mathematics, item 1	2.257	1.287	0.615	-0.785
Predisposition toward mathematics, item 2	2.094	1.130	0.735	-0.334
Predisposition toward mathematics, item 3	2.000	1.267	1.106	0.074
Predisposition toward mathematics, item 4	2.125	1.142	0.951	-0.517
Predisposition toward mathematics, item 5	2.260	1.249	0.689	-0.516
Predisposition toward mathematics, item 6	1.969	1.227	1.186	0.304
School performance in mathematics, year 1	5.994	0.757	-0.861	0.199
School performance in mathematics, year 2	5.808	0.789	-0.613	-0.317
School performance in mathematics, year 3	5.662	0.802	-0.471	-0.537
School performance in mathematics, year 4	5.525	0.897	-0.784	0.787

## Results

### Structural Equations Model of the Early Mathematical Competencies (EMCs) Variable

Initially, a univariate descriptive statistical analysis of the variants examined in this study was developed (see **Table 1**).

**FIGURE 1:**
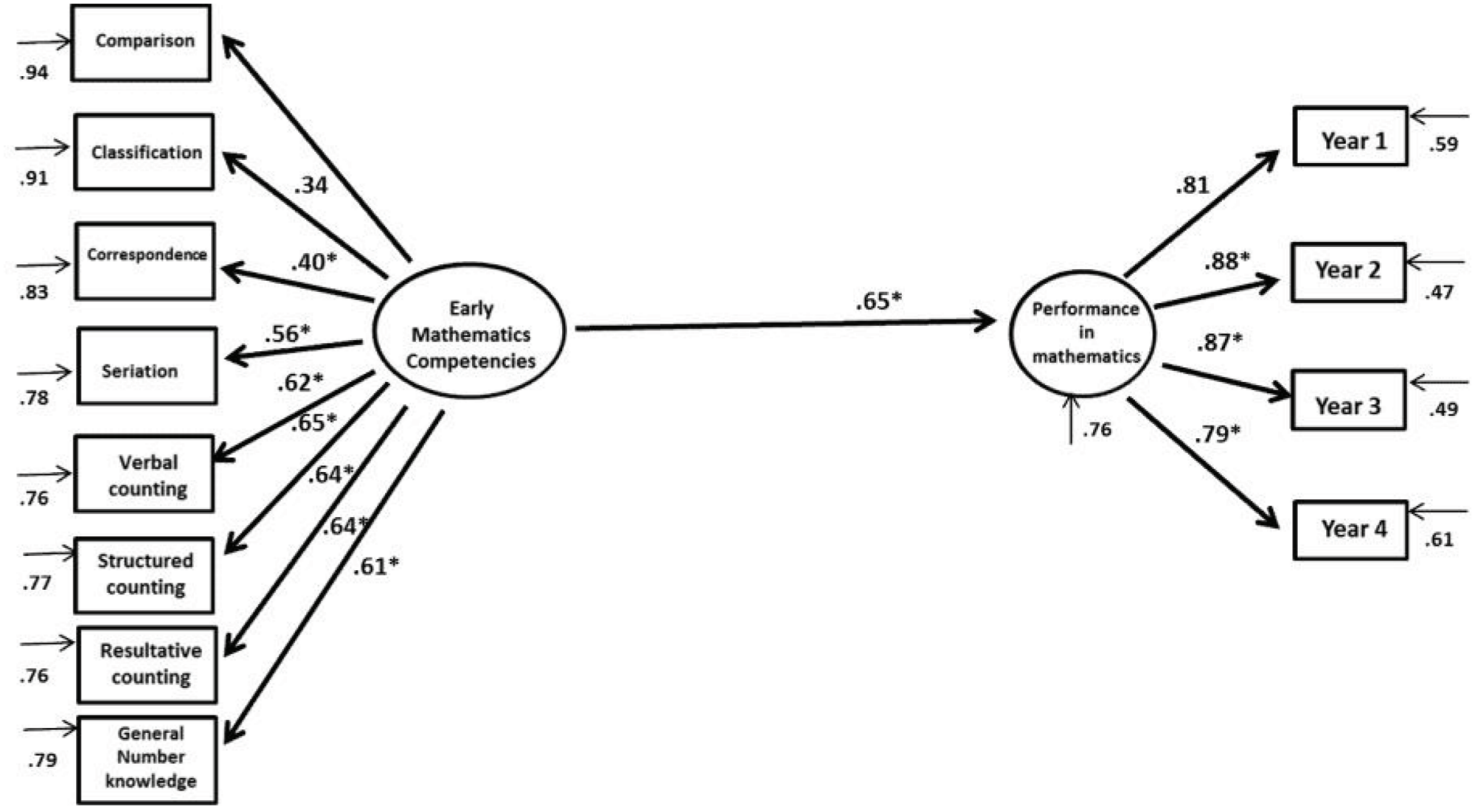
**Structural equations model of the interaction of early mathematical competencies (EMCs) with academic performance in mathematics**.

Considering the first objective of this study, we developed an explanatory model that took into account solely the effect of the latent EMC variables on academic performance. In **Figure 1**, we can see a graphic solution of this model, where EMCs explain 42.1% of the variability of the means of student grades observed during the analyzed period of 4 years. The model’s adjustment indices were fit: χ*^2^SB* = 115.86, *p* = 0.00002, *CFI* = 0.974, *NNFI* = 0.968, *RMSEA* = 0.043, *IC*(0.032–0.054).

### Structural Equations Model of Cognitive and Predisposition Variables

**FIGURE 2:**
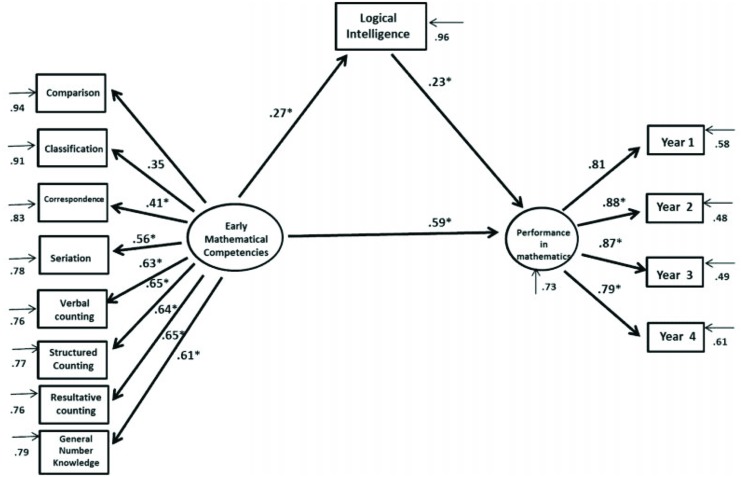
**Structural equations model proposed for the complex interaction between EMC variables and logical intelligence concerning academic performance in mathematics**.

With respect to the second objective of this research, a structural equations model to analyze the influence of logical intelligence on the previous model was developed. In this way, the second model indicates the interrelationship between EMCs and the level of logical intelligence with respect to students’ grade point averages in the subject of mathematics (see **Figure 2**).

The model’s adjustment indices were the following: χ^2^*SB* = 133.38, *p* = 0.000, *CFI* = 0.973, *NNFI* = 0.966, *RMSEA* = 0.042, *IC*(0.032–0.052) and they showed optimal fitting. Standard regression coefficients indicate a direct and positive influence of EMCs on students’ grade point averages in mathematics (β = 0.59; *p* < 0.001), and, also, on the level of logical intelligence (β = 0.27; *p* < 0.001). Likewise, a positive and significant relationship of logical intelligence on mathematics performance was observed (β = 0.23; *p* < 0.001). This model predicts 47.1% of the variance of students’ grade point averages in the subject of mathematics.

**FIGURE 3:**
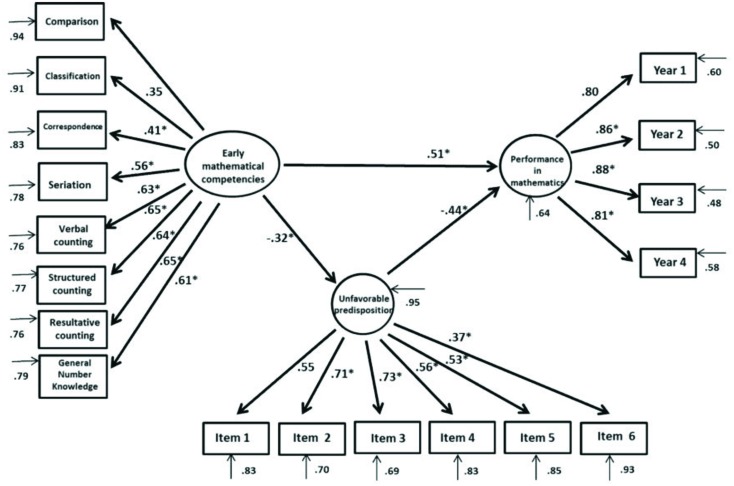
**Structural equations model of the complex interaction between EMCs and unfavorable predisposition toward mathematics with respect to performance in mathematics**.

Subsequently, the influence of predisposition toward mathematics for academic performance in mathematics was assessed (**Figure 3**). The model’s adjustment indices were χ^2^*SB* = 291.52, *p* = 0.000, *CFI* = 0.953, *NNFI* = 0.945, *RMSEA* = 0.044, *IC*(0.037–0.050), showing optimal fitting. Standardized regression coefficients indicate that EMCs have a direct and positive influence on students’ grade point averages in mathematics (β = 0.51; *p* < 0.001) and that they have an inverse and significant relation with an unfavorable predisposition toward mathematics (β = -0.32; *p* < 0.001). Likewise, an unfavorable predisposition toward mathematics has a negative and significant relation with academic performance in mathematics (β = -0.44; *p* < 0.001). This model predicts 59.7% of the variance in students’ grade point averages in the subject of mathematics.

**FIGURE 4:**
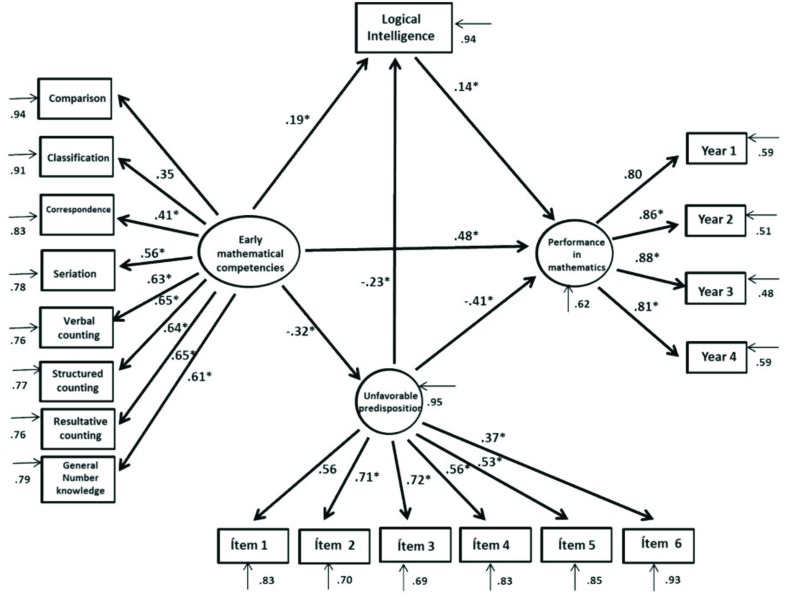
**Structural equations model of the complex interaction between the variables of early mathematical competencies, unfavorable predisposition toward mathematics, and logical intelligence with respect to performance in mathematics**.

Lastly, when assessing the last hypothetical model proposed (**Figure 4**), we can see that this model shows fit adjustment indices χ^2^*SB* = 314.32, *p* = 0.000, *CFI* = 0.953, *NNFI* = 0.945, *RMSEA* = 0.042, *IC*(0.036–0.049). Standardized regression coefficients indicated that EMCs have a positive and significant effect on academic performance in mathematics (β = 0.48; *p* < 0.001). Meanwhile, predisposition toward mathematics has an inverse influence on students’ grade point averages (β = -0.41; *p* < 0.001), and logical intelligence has a positive correlation with students’ grade point averages in mathematics (β = 0.14; *p* < 0.05). Likewise, we observed that EMCs have a positive and significant relationship with logical intelligence (β = 0.19; *p* < 0.05) and a negative relation with an unfavorable predisposition toward mathematics (β = -0.32; *p* < 0.01). Also, an unfavorable predisposition toward mathematics has a negative and significant correlation with logical intelligence (β = -0.23; *p* < 0.05). The model’s adjustment indices were fit: χ^2^*SB* = 329.03, *p* = 0.001, *CFI* = 0.953, *GFI* = 0.945, *NNFI* = 0.945, *RMSEA* = 0.042, *IC*(0.036-0.049). The variables included in this model explain 61.3% of the variability in students’ academic results in the subject of mathematics.

## Discussion

According to the results of this study, there is enough evidence to validate the initial hypothesis regarding the predictive character of EMCs in respect to mathematics performance [H1]. The relevance of this study is its longitudinal methodology, which allowed the researchers to monitor participants’ performance through a period of 4 academic years of primary school. This shaped the predictive power of the EMC construct and also contributes to the several findings regarding the fact that preschool students’ early mathematics performance is a strong predictor of later mathematics achievement (Toll and Van Luit, 2014). These results are also in line with the results from Passolunghi and Lanfranchi (2012) who found that EMCs greatly predict learning achievement in mathematics in the first level of primary school.

Furthermore, students’ previous levels of EMCs achievement in counting and numerical tasks proved to have a strong relationship with their school achievement during their first years of primary education; similar results were also found in a study conducted by Navarro et al. (2012) in Spain. The predictive role of EMCs replicates the results from studies about logical, Piagetian ability tasks involved in seriation and classification tasks, or numerical counting tasks and their effect on performance in mathematics (Nunes et al., 2007; Desoete et al., 2009). Likewise, these findings also replicate the results of research from Kroesbergen et al. (2012) and Xenidou-Dervou et al. (2013), who examined the role of number sense, number magnitude representation and number comparison. According to these authors, these variables also greatly contribute to explain the variation in mathematics performance during the first years of primary education.

The predictive potential of EMCs is particularly relevant because this predictive character does not diminish with time, especially in the case of mathematical domains linked to solving problems in different contexts (Jordan et al., 2010). Furthermore, a great deficiency in these abilities predicts a poor performance in tasks that imply symbolic manipulation of numbers, which could also explain the relationship between dyscalculia and the alteration of this early number sense (Piazza et al., 2010).

When contrasted, the theoretical model corroborated the importance of students’ predisposition toward mathematics, as this variable had a high relative weight regarding academic performance for this subject. Likewise, an unfavorable predisposition toward mathematics is inversely related with performance in the discipline. This emotional factor may be of particular importance if we consider that mathematics is usually a subject toward which students manifest a negative predisposition or attitude for different reasons, for example, due to the teaching method used, parents’ expectations, the teaching style of the instructor, students’ own beliefs, or the influence of stereotypes based on social and cultural factors (Vandecandelaere et al., 2012; Yaratan and Kasapoǧlu, 2012). Likewise, it is important to note that when a student chooses to follow a career in a particular field of studies, like mathematics, their beliefs about their attitude toward the field may actually be more important than what they actually do feel toward the field of studies (Goetz et al., 2014).

This study also goes along with other studies that place emphasis on the modulating and incremental role of motivational variables to explain academic performance, specifically when their effect is modelled in combination with attitude or cognitive variables (Miñano and Castejón, 2011; Jansen et al., 2013). In the case of our study, EMCs along with predisposition toward mathematics and logical intelligence explained an important percentage—around 61%, of the variability observed in students’ school performance in mathematics. In this model, predisposition toward mathematics had a modulating role that was capable of mitigating the direct effect of the cognitive variables analyzed, like, for example, the effect of the initial mathematical competencies assessed in relation to performance in mathematics. A similar study that used logistic regression analysis also established that the participants with more motivation toward mathematics, who had less discipline problems and achievement scores over the mean on mathematics tests, had a high probability of reaching a high to a normal average performance in the subject (Cerda and Pérez, 2014; Cerda et al., in press b).

This also evidences the close relationship with experimental research studies that point out that when a pre-established success rate is high, students face a higher number of problems, which, at the same time, favorably impacts their performance in mathematics (Jansen et al., 2013). Seen from an experimental perspective, one could also infer a higher level of acquisition of EMCs, and, therefore, a higher correct resolution rate in tasks similar to the ones involved in the eight analyzed dimensions. Likewise, this could greatly influence the valence of the predisposition variable. Accordingly, it has been observed that students less skilled in mathematics tend to choose activities that are less cognitively demanding, like simple motor tasks (Edens and Potter, 2013).

On the other hand, mathematics is a subject from the school curriculum that requires superior cognitive abilities as well as interconnected and cumulative knowledge, which is progressively put into practice. This is especially relevant when other studies have established that the subjects who have a good performance in mathematics tend to be those that have overall good academic performance (Cerda et al., in press c). Consequently, when an individual is not able to progress in their learning, maybe he or she begins to incubate a self-perception of failure or incompetence, which causes rejection, anxiety, frustration, or emotional blocks in subsequent experiences involving mathematical tasks (Molera, 2012); likewise, this frustration can also spread to the other areas of learning. In fact, students with low performance in mathematics show from the beginning a tendency to avoid schoolwork, while successful students tend to improve more than their peers (Hirvonen et al., 2012; Kikas et al., 2014). This could also partially explain the reason why secondary students show a lower predisposition toward mathematics than the groups of elementary school students. The first group has probably experienced a wider range of negative experiences in this subject. This emotional predisposition probably interacts reciprocally with the attitude from the teachers of this subject or with the amount of support given by the teachers to the students (Marchisa, 2011).

Future perspective about the relationship between emotional factors and mathematical learning will require long-term studies. After having conducted this study, we consider that a more comprehensive longitudinal assessment of the emotional and complex attitudes toward mathematical learning would shed light about the relationship between these two variables, which could create opportunities for the design of teaching and learning strategies for a school subject on which there is a high rate of academic failure in our society.

## Conflict of Interest Statement

The authors declare that the research was conducted in the absence of any commercial or financial relationships that could be construed as a potential conflict of interest.
